# Supplementary material for: Chromothripsis during telomere crisis is independent of NHEJ, and consistent with a replicative origin
Source: Genome Res. 2019 May;29(5):737–49. doi: 10.1101/gr.240705.118 (PMC6499312; doi:10.1101/gr.240705.118)
Supplement: Supplemental Material [file supp_gr.240705.118_Supplemental_file_1.zip › contigs/annotated_contigs/DB78/contig.2.DB78_length_727_mean_cov_8.35625859697.docx]

**DB78_length_727_mean_cov_8.35625859697**

```
CCCATGGTGGGGAGCGGCCGGCTTGGAGCCCCTGTGGGACGGAGTGGAGGTGGAGCAAGTGCCCGGAGCTCTCGGCCATCATGTGCGAA
 >chr16:863402-863742 + E=9e-190
TGTTTTGCTCCGTGCTGACGCCTCCCTCGGCACCGTCCTGTCTGTGTTGTGGACTGGGCAGCTCTCACGGGGTTGGGCGCTTCTGCCCC

CGGGGGACGCTGGCCGGCATCTGGAGACGTCTGTGATCTCCGCTGGTGTTGCCGCAGGCATCTGGCTGGTGGAGCCCGGGGAGGCCGCC

CAGGACCCTGCGACCCGCAGGACCGCCACGCCGCGGAGGACGGCCAGCCCCGAACCCCCAGCGCCCGGGG|CAC|GGGCAAGGCGGTCC
                                                                       >chr16:779308-77
CCGCCTTCGTCCTGGAGGCGCTATGCAAGGAGCGGGCGCGCTTCGAACAGGCAGGCGAGCCGCGGCAGCCACAGGCGCGGGCCCTCCTT
9698 + E=8e-214
GCGCTGCAGTGGGGGCTCCTGAGTGCAGCCCCACACGACTGAGCCACCCAGACCCTCACAGGGCTGGGGCCTGCGTCCCTCCTCGTGAC

CTCGGCCAGCTGGACCCACATGAGGGCCACCTCTGGAACCTTCTTCGAGGCCCTGGCCAGCCATCTGCCCAGCCTCGGAGGGTGGGCAA

CCTGGTGCTTCCCGGGTGGACACACAGGACCACTCAGTGGGGCCTGTGTGGGCGCCGAGACCTGGGTGTCTGGGAAGTGGGGCACACGG

GGCCTCCGAACTATGAA
```
